# Role of PD-L1 in the Pathogenesis of Pre-Eclampsia and Its Association with Adverse Fetal Outcomes

**DOI:** 10.5146/tjpath.2025.13658

**Published:** 2026-05-30

**Authors:** Sonal Tripathi, Kachnar Varma, Vatsala Misra

**Affiliations:** Department of Pathology, Moti Lal Nehru Medical College, PRAYAGRAJ, INDIA

**Keywords:** Pre-eclampsia, PD-L1, Placental immunology, Immune dysregulation

## Abstract

Objective: Preeclampsia is a pregnancy-specific disorder characterized by impaired maternal-fetal immune tolerance. The maternal immune system plays a crucial role in maintaining pregnancy, and its dysfunction is believed to contribute to preeclampsia. Immune checkpoint molecules such as programmed cell death protein 1 (PD-1) and its ligand, programmed death ligand 1 (PD-L1), may play a key role in this process.

This study evaluated PD-L1 expression in the placentae of patients with pre-eclampsia (PE) and eclampsia (EC). We also compared PD-L1 expression with histomorphological features and fetal outcomes.

Material and Methods: A prospective case-control study was conducted, including fifty pre-eclampsia cases, twenty-five eclampsia cases, and twenty-five normal pregnancy controls. Detailed clinicopathological data, histomorphological features of the placenta, and fetal outcomes were collected. PD-L1 expression was assessed using immunohistochemistry, with a semi-quantitative scoring system. The relationship between PD-L1 expression, histopathological scores, and fetal outcomes was also evaluated.

Results: In this study, a lower expression of PD-L1 was observed in pre-eclampsia and eclampsia as compared to a normal pregnancy. Adverse fetal outcomes were associated with lower PD-L1 expression and with reduced placental weight and high histopathological scores (>5).

Conclusion: Lower PD-L1 expression was observed in pre-eclampsia and eclampsia compared to normal pregnancies. Reduced PD-L1 expression correlated with histomorphological changes in the placenta and adverse fetal outcomes.

## Introduction

Preeclampsia (PE) is a pregnancy-specific disorder characterized by impaired materno-fetal immune tolerance (1). These immune-mediated conditions contribute significantly to maternal and fetal morbidity and mortality. Adverse fetal outcomes associated with PE include prematurity, intrauterine growth restriction, and stillbirth. Although the exact etiology is complex, impaired maternal immune responses are believed to play a pivotal role in its pathogenesis (2,3).

The programmed death-1 (PD-1) receptor and its ligand, PD-L1, form an important molecular pathway involved in immune regulation during pregnancy. This PD-1/PD-L1 axis helps maintain maternal tolerance to the fetus by promoting the development of regulatory T (Treg) cells and inhibiting effector T cell responses. Dysfunction in this pathway, particularly reduced expression of PD-L1, may contribute to the pathogenesis of PE and eclampsia (EC) by allowing overactivation of T cells (4,5).

While the role of PD-L1 in maintaining normal pregnancy has been established, few studies have specifically evaluated its expression in PE and EC. This study aims to correlate the expression of PD-L1 in the placentae of patients with PE and EC and to investigate its association with histomorphological features and fetal outcomes.

## Materials and Methods

This prospective case-control study was conducted at a tertiary care center over two years, focusing on women diagnosed with PE and EC. Placental tissue from these patients was compared with placental tissue from healthy controls.

**Inclusion Criteria**

**PE:** Female patients aged ≥18 years with a blood pressure of more than or equal to 140/80 mmHg and proteinuria (more than or equal to 0.3 g/24 hours) after 20 weeks of pregnancy.

**EC:** Patients diagnosed with PE complicated by seizures.

**Control**: Healthy pregnant women aged ≥18 with normal, full-term deliveries.

**Exclusion Criteria**

Patients with co-morbidities such as chronic hypertension, diabetes mellitus, hepatitis B and C, or HIV were excluded. The study included 100 placental tissues: 50 from PE patients, 25 from EC patients, and 25 from controls. Detailed clinicopathological data were collected after obtaining informed consent from participants.

**Histopathological Analysis**

**Figure 1:**
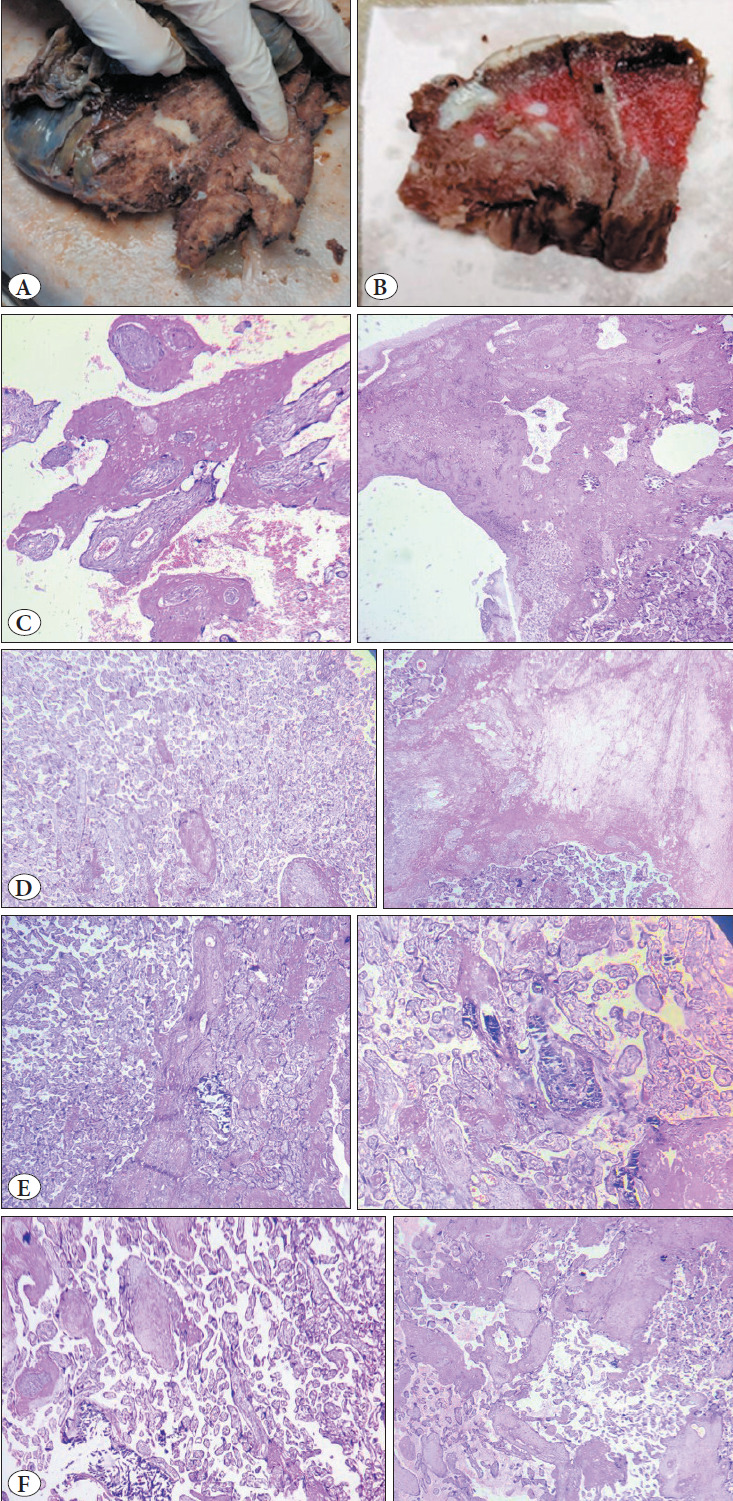
Gross and histopathological features A) Placenta with areas of infarction. B) Focal areas of Calcification. C) on H & E areas of fibrin deposition (Intravillous (score 1) v/s intravillous and perivillous (score 2) on 40X. D) On H & E, material floor infarction seen in <50% of area (score 1) v/s >50% of area (score 2)on 40X. E) on H & E areas of calcification seen in a single area( score 1) v/s multiple areas (score 2) on 40X. F) on H & E areas of thickening of basement membrane (score 1) v/s hyalinization

Placental tissue was grossly and microscopically examined according to standard protocols (6). The weight and thickness of the placenta were recorded, and areas of infarction Figure 1 and calcification Figure 1 were noted. Microscopic lesions were scored using a semi-quantitative scoring system (7):

**Figure 2:**
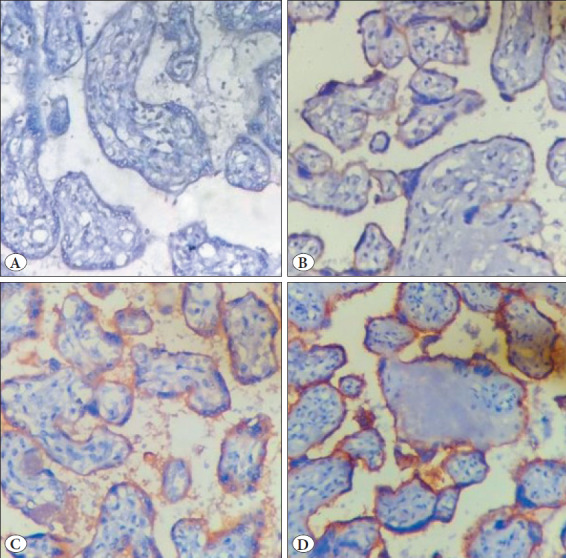
PD-L1 IHC.A) [Score 0]: 0-10% of the circumference of villi shows PD-L1 expression on 40x. B) [Score 1]: 10-50% of the circumference of villi shows PD-L1 expression on 40x. C) [Score 2]: 50-90% of the circumference of villi shows PDL1expression on 40x . D) [Score 3]: >90% of circumference of villi shows PD-L1 expression on 40x.

**Fibrin Deposition** Figure 1 **:**

Score 0: Absent

Score 1: Intravillous/Perivillous

Score 2: Intravillous and Perivillous

**Maternal Floor Infarction** Figure 1 **:**

Score 0: Absent (less than 5% infarcted area)

Score 1: Single area (more than 5% infarcted in a single area)

Score 2: Multiple area (more than 5% infarcted in more than one area)

**Calcification** Figure 1 **:**

Score 0: Absent

Score 1: Focal

Score 2: Diffuse

**Syncytial Knots:**

Score 0: Absent

Score 1: Present

**Villous Basement Membrane Thickening** Figure 1 **:**

Score 0: Absent (< 3% thickening)

Score 1: Thickened (> 3% thickening)

Score 2: Hyalinized

**Immunohistochemistry and Semi-Quantitative Scoring of PD-L1 Expression**

Immunohistochemistry (IHC) was performed to evaluate PD-L1 expression in placental tissue. The tissue blocks from placental biopsies were selected for analysis, focusing on the villi and decidua basalis. Monoclonal Rabbit Anti-PD-L1 (clone RBT-PDL1, Bio SB, USA) was the primary antibody, diluted in PBS with 1% BSA and 0.09% sodium azide.

Antigen retrieval was performed using a microwave technique with citrate buffer (pH 6.0), followed by hydrogen peroxide treatment to block endogenous peroxidase. After incubating with the primary antibody for 1 hour, the secondary antibody was applied for 45 minutes. Tonsil tissue was used as a positive control, while negative controls omitted the primary antibody. Slides were counterstained with hematoxylin and mounted.

PD-L1 expression was assessed by examining the membranous staining on the outer surface of syncytiotrophoblasts (8). Ten random villi were selected from the fetomaternal interface at 4x magnification and the extent of staining were analyzed as shown in Table 1. The scoring system was as follows:

**Table 1:** Semi-quantitative scoring for PD-L1 expression (7).

**PD-L1 Score**	**Percentage of the circumference of villi-stained**
0	0-10 % (Figure 2)
1	10-50 % (Figure 2)
2	50-90 % (Figure 2)
3	>90 % (Figure 2)

**Score 0** (Figure 2): 0-10% (circumference of villi-stained)

**Score 1** (Figure 2): 10-50% (circumference of villi-stained)

**Score 2** (Figure 2): 50-90 %(circumference of villi-stained)

**Score 3** (Figure 2): >90%(circumference of villi-stained)

An average score from ten villi was calculated, and scores 0-1 were considered low expression, while scores 2-3 were considered high expression.

PD-L1 expression was correlated in patients with PE, EC, and controls. Additionally, PD-L1 expression showed correlations with histopathological features and fetal outcomes, as detailed in Table 2.

**Table 2:** Association of PD-L1 score with Clinicopathological parameters.

**PD-L1 score**	**Fetal** **outcome**		**Placental** **weight**		**Histopath score**	
** **	**Normal**	**Adverse**	**<400 gms**	**>400 gms**	**<5**	**>5**
0	10	30	30	4	33	6
1	4	20	21	3	20	6
2	8	8	13	12	12	6
3	18	2	5	12	4	13
**p-value**	0.827		0.001		0.001	

## Statistical Analysis

Data analysis was performed using one-way analysis of variance (ANOVA) to compare PD-L1 expression scores between PE, EC, and control groups. The correlation between PD-L1 expression, histopathological features, and fetal outcomes were also assessed. A p-value of less than 0.05 was considered statistically significant.

## Results

The clinicopathological presentation of patients and controls is summarized in Table 3.

**Table 3:** Clinicopathological Characteristics of case and control.

**Characteristics**	N.P* **n=25 (%)**	P.E** **n= 50 (%)**	**E.C‡** **n=25 (%)**
**Age (Years)** <20 21-25 26-30 31-35	- 3 (12) 14 (56) 6 (24) 2 (8)	- 8 (16) 22 (44) 15 (30) 5 (10)	- 4 (16) 12 (48) 6 (24) 3 (12)
**Gestational Age(weeks)** 25-30 30-35 35-40	- 2 (8) 4 (16) 19 (76)	- 7 (14) 28 (56) 15 (30)	- 7 (28) 10 (40) 8 (32)
**Parity** Multigravida Primigravida	- 14 (56) 11 (44)	- 26 (52) 24 (48)	- 11 (44) 14 (56)
**Weight of placenta** <400 gms >400 gms	- 9 (36) 16 (64)	- 28 (56) 22 (44)	- 17 (68) 8 (32)
**Placental thickness** <2 cm >2 cm	- 9 (36) 16 (64)	- 26 (52) 24 (48)	- 16 (64) 8 (32)

*N.P: Normal Pregnancy, **P.E: Pre-eclampsia, **‡E.C:** Eclampsia

In this study, the age distribution between the three groups—Normal Pregnancy (NP), Pre-Eclampsia (PE), and Eclampsia (EC)—did not show any significant differences. The majority of participants in all three groups were in the 21-25 years age range, with NP and PE cases being predominantly in this age group, followed by EC.

The gestational age at the time of delivery was significantly different between the groups. In the NP group, most pregnancies were carried to 35-40 weeks gestation, whereas PE and EC cases had a higher proportion of pregnancies at 25-35 weeks gestation, indicating a trend towards preterm delivery in these conditions.

There was no significant difference in parity between the groups, with both primigravida and multigravida patients distributed relatively evenly across the three groups.

Regarding placental characteristics, there was a significant difference in placental weight across the groups. “In the PE and EC groups, the majority of the placentas weighed less than 400 grams, and in contrast, only 36% of placentas in the NP group weighed less than 400 grams.

Similarly, placental thickness was significantly lower in both PE and EC groups than NP.

Immunohistochemical analysis of PD-L1 expression as seen in Table 4 revealed a significant difference between the groups. In the PE and EC groups, low PD-L1 expression (scores 0 and 1) was seen in the majority of cases (80% and 88%, respectively), whereas the NP group showed predominantly high PD-L1 expression (scores 2 and 3) in 84% of cases. The difference in PD-L1 expression between the groups was statistically significant (p-value 0.001), with normal pregnancies showing higher expression than the diseased groups.

The correlation between PD-L1 expression and various clinical and histopathological parameters was also assessed as seen in Table 4. Placental weight showed a significant association with PD-L1 expression. Cases with low placental weight (less than 400 grams) had predominantly low PD-L1 expression (scores 0 and 1), while those with normal placental weight (less than 400 grams) showed higher PD-L1 expression (scores 2 and 3) (p-value <0.05).

Although adverse fetal outcomes (low birth weight and stillbirth) were more common in cases with low PD-L1 expression, this association was not statistically significant (p-value 0.827).

However, a trend was observed where higher PD-L1 expression was linked to better fetal outcomes, with 19% of cases with scores 2 and 12% with a score of 3 showing normal fetal outcomes, compared to lower fetal survival rates in cases with scores 0 and 1.

In terms of histopathological severity, a higher histopathological score (greater than 5) was associated with low PD-L1 expression (33% in score 0, 20% in score 1), while higher PD-L1 expression was observed in cases with a lower histopathological score (6% in score 2, 13% in score 3) (p = 0.01). This suggests that low PD-L1 expression may be associated with more severe placental pathology.

## Discussion

Preeclampsia (PE) and eclampsia (EC) are major immune-mediated complications with pregnancy, with a significant burden on maternal and fetal health, particularly in the Indian subcontinent, where the incidence ranges from 2-10%. These conditions are multifactorial in origin, with maternal immune dysregulation being a key contributor to their pathogenesis. The immune checkpoint molecule, PD-L1, plays a crucial role in maintaining immune tolerance during pregnancy, and this study explored its role in the pathogenesis of PE and EC, correlating PD-L1 expression with fetal outcomes and placental histopathological findings.

The study identified that the majority of affected women were aged 21-25 years, followed by the 26-30 years’ age group. This observation aligns with evidence suggesting that vascular aging and increased arterial stiffness contribute to endothelial dysfunction, a characteristic feature of PE (9–12). Additionally, primiparity emerged as a significant risk factor for PE, likely due to the first-time exposure to fetal antigens from the chorionic villi, which challenges maternal immunological adaptation (13,14).

Gestational age was significantly impacted, with preterm delivery (less than 37 weeks) frequently observed in PE and EC cases. This finding corroborates earlier studies suggesting that preeclampsia leads to preterm delivery rather than being caused by it (15,16). Adverse fetal outcomes, such as fetal growth restriction (FGR), low birth weight (LBW), and stillbirths, were more prevalent in the case group. LBW was observed in 44% of PE and 48% of EC cases, similar to 40% in normotensive pregnancies. However, the incidence of stillbirths was three times higher in PE and EC cases than controls, underscoring the profound impact of placental insufficiency and maternal systemic inflammatory responses (17,18).

Placental pathology revealed significant gross and histopathological changes in PE and EC.

Placentas in the case group often weighed less than 400 g (56% in PE and 64% in EC) and exhibited reduced thickness (less than 2.0 cm) (19). Histomorphological findings included abnormal vasculature, parenchymal infarction, intervillous and perivillous fibrin deposition, syncytial knotting, and villous basement membrane thickening. These findings reflect severe placental malperfusion, correlating with the severity of maternal hypertension (20).

The PD-L1 pathway emerged as a crucial mediator in terminating immune responses and inducing immune tolerance. PD-L1 promotes regulatory T cell (Treg) development while inhibiting effector T cell responses, specifically Th17 immunity, contributing to exaggerated systemic inflammation (21–23).

Apart from autoimmune disorders, the PD-1/PD-L1 pathway also establishes maternal-fetal tolerance by promoting the Treg/Th17 balance. It has been reported that pregnant mice treated with anti-PD-L1 blocking anti-body lose their embryos. A deficiency in PD-L1 has been associated with an increased frequency of fetal resorption and decreased fetal survival (24). However, the precise role of the PD-1/PD-L1 pathway in PE is unclear, thus this study was undertaken to understand the role of PD-1/PD-L1 in the pathogenesis of PE.

Immunohistochemistry analysis of PD-L1 expression in PE and EC cases revealed a predominant staining pattern on the membranous side of the chorionic villi. Also, a semi-quantitative scoring system of PD-L1 was used, and PD-L1 expression was evaluated by analyzing the percentage of the circumference of the villi stained, which was scored from 0-3 (8). Comparison of PD-L1 expression in the case groups (PE and EC) to the control group (NP) as done using statistical analysis as shown in Table 4.

**Table 4:** Correlation of PD-L1 expression in cases of Pre-eclampsia and Eclampsia.

**PDL-1 Score**	**Percentage of the circumference of villi stained**	N.P.* **n=25 (%)**	P.E.** **n=50 (%)**	**E.C.‡** **n=25 (%)**	**p-value**	**Statistical Significance**
**0**	0-10%	1 (4)	12 (24)	14 (56)	0.001	**Significant**
**1**	10-50%	3 (12)	28 (56)	8 (32)		
**2**	50-90%	11 (44)	8 (16)	2 (8)		
**3**	>90%	10 (40)	2 (4)	1 (4)		

*N.P: Normal Pregnancy, **P.E: Pre-eclampsia, **‡E.C:** Eclampsia

Correlation analyses unveiled associations between lower PD-L1 expression and adverse fetal outcomes, as well as specific histopathological parameters as seen in Table 2. The study suggested a potential correlation between gross and histopathological features of PE and PD-L1 expression, indicating a disrupted balance between immunotolerance and immunoactivation. Moreover, murine model findings supported the crucial role of PD-1/PD-L1 in normal pregnancy and fetal outcomes. Variable PD-L1 expression has been seen in patients with PE. Some authors have found reduced expression, whereas others have found increased expression of PD-L1 in cases of PE (24,25). Further investigation is warranted to delineate the role of programmed death ligand 1 (PD-L1) in pre-eclampsia (PE). Limitations of our study include a smaller sample size and reliance on immunohistochemistry (IHC) for PD-L1 expression evaluation. Other sensitive techniques like polymerase chain reaction (PCR) and flow cytometry could enhance precision.

Our study uniquely correlated histopathological parameters with PD-L1 expression. A significant association was found between lower PD-L1 expression and histopathological features, including low placental weight, fibrin deposition, maternal floor infarction, and villous basement membrane thickening Table 3. This novel approach highlights a potential link between gross and histopathological manifestations in PE. The disrupted balance between immunotolerance and immune activation signifies immune regulation failure, exacerbating inflammatory responses and PE (26). Additionally, these factors may contribute to placental malperfusion, manifesting as histomorphological changes observed in our study. PD-L1 expression was further correlated with adverse fetal outcomes, revealing that low PD-L1 expression is associated with adverse outcomes due to abnormal maternal inflammatory responses. Although limited human studies were found, murine models demonstrated a higher rate of fetal growth restriction and fetal resorption in pre-eclamptic rats. Treating these rats with PD-L1-Fc corrected these developmental aberrations, reinforcing the crucial role of PD-1/PD-L1 in normal pregnancy and fetal outcomes.

In conclusion, our study highlights the pivotal role of PD-L1 in pre-eclampsia pathogenesis and adverse fetal outcomes (27,28). Larger studies are warranted to explore PD-L1 pathway activators like PD-L1-Fc as potential therapeutic targets in PE treatment (27,29,30). This study emphasizes the significance of standardized reporting using a uniform scoring system for placental pathologies associated with uteroplacental insufficiency, such as pre-eclampsia.

This study emphasizes the importance of PD-L1 in the pathogenesis of PE and its association with adverse fetal and placental outcomes. The findings suggest that restoring PD-L1 function could be a potential therapeutic strategy. Larger studies incorporating advanced techniques such as polymerase chain reaction (PCR) and flow cytometry are needed to validate these findings and refine our understanding of PD-L1’s role. Additionally, standardization of placental pathology reporting using uniform scoring systems would enhance clinical relevance and facilitate the exploration of PD-L1 activators, such as PD-L1-Fc, as therapeutic options in the management of PE. The study underscores the disrupted balance between immunotolerance and immune activation in PE, providing insights into its pathophysiology and potential interventions.

## Conclusion

This study substantiates notable disparities in both gross and histomorphological characteristics between pre-eclampsia/eclampsia and normal pregnancies. Implementing a standardized scoring system is advocated for precise reporting of placental pathologies, particularly those linked to uteroplacental insufficiency, such as pre-eclampsia. Additionally, our findings underscore the pivotal involvement of programmed death ligand 1 (PD-L1) in the pathogenesis of pre-eclampsia, prompting a call for further investigations to scrutinize and authenticate its role. Prospective studies are warranted to assess the efficacy of PD-L1 pathway activators, including PD-L1-Fc, as potential therapeutic targets in treating pre-eclampsia. 

## Conflict of Interest

The authors have no conflicts of interest to declare.
